# Prokaryotic taxonomy and functional diversity assessment of different sequencing platform in a hyper-arid Gobi soil in Xinjiang Turpan Basin, China

**DOI:** 10.3389/fmicb.2023.1211915

**Published:** 2023-11-14

**Authors:** Zhidong Zhang, Jing Zhu, Osman Ghenijan, Jianwei Chen, Yuxian Wang, Ling Jiang

**Affiliations:** ^1^Xinjiang Key Laboratory of Special Environmental Microbiology, Institute of Applied Microbiology, Xinjiang Academy of Agricultural Sciences, Urumqi, China; ^2^BGI Research, Qingdao, China; ^3^College of Food Science and Light Industry, Nanjing Tech University, Nanjing, China; ^4^State Key Laboratory of Materials-Oriented Chemical Engineering, Nanjing Tech University, Nanjing, China

**Keywords:** Turpan Basin, next generation sequencing, hyper-arid soil, prokaryotic diversity, community assembly

## Abstract

Turpan Basin located in the eastern Xinjiang is a typical arid inland basin with extremely scarce water resources and a fragile ecosystem. Prokaryotic communities with unique genetic and physiological modifications can survive and function in such harsh environments, offering diverse microbial resources. However, numerous microbes can enter the viable but non-culturable state because of drought stress in the desert soil. In this work, next generation sequencing (NGS) technology based on DNA nanoball sequencing platform (DNBSEQ-G400) and sequencing-by-synthesis platform (NovaSeq 6000) were applied to analyze the prokaryotic diversity in three hyper-arid Gobi soils from Flaming Mountain, Toksun, and Kumtag. The comparison between two platforms indicated that DNBSEQ-G400 had better repeatability and could better reflect the prokaryotic community of this hyper-arid region. The diversity analysis based on DNBSEQ-G400 identified a total of 36 bacterial phyla, including *Pseudomonadota*, *Bacteroidota*, *Bacillota*, *Actinomycetota*, *Methanobacteriota*, *Acidobacteriota*, *Nitrososphaerota*, and *Planctomycetota*. The environmental factors, including soluble salt, available potassium, total nitrogen, and organic matter, were positively correlated with the abundance of most prokaryote. In addition, the prokaryotic community assembly in hyper-arid soil was well described by neutral-based models, indicating that the community assembly was mainly controlled by stochastic processes. Finally, the phylogenetic analysis of *Actinomycetota* proved that such extremophiles played an important role in the ecosystems they colonize. Overall, our result provides a reference for choosing the appropriate sequencing platform and a perspective for the utilization of soil microbial resources from hyper-arid regions.

## Introduction

Climate extremes have a significant impact on the global ecology, which will be further aggravated continuously (Zhang Q. et al., 2021; Zhang Y. et al., 2021). Among them, dryland zones are one of the typical ecologically fragile regions in the global ecosystem. It has been reported that dryland zones, including semi-arid, arid and hyper-arid areas, account for 40% of Earth’s terrestrial surface (Yao et al., 2020). Within dryland conditions, hyper-arid areas have the driest environment characterized by extremely low mean annual precipitation, extremely high ultraviolet (UV) irradiation and an atmospheric relative humidity that usually drops to zero in the afternoon, resulting in barren land without vegetation (Mao et al., 2018; Belov et al., 2019). Although the living conditions in hyper-arid areas are extremely harsh, they have rich and diversified microbial resources, which are an important part of desert ecosystems (Zhang et al., 2020). Therefore, it is of great significance to comprehensively and deeply understand the impact of high temperature and drought on microbial behavior and ecosystem functions.

Xinjiang Uygur Autonomous Region constitutes more than 80% of the arid zone in northwestern China (He et al., 2023). The Turpan Basin, located in eastern Xinjiang, is a typical arid inland basin with extremely scarce water resources and a fragile ecosystem (Huang et al., 2021). The extreme evaporation combined with low precipitation rates results in the hyper-arid continental climate of this zone. In this extreme environment, only highly drought-tolerant oligotrophic microbial species can survive, and even these are discontinuously distributed. Nevertheless, a series of recent studies demonstrated that the hyper-arid soils found in these zones can support complex microbial assemblages (Zhang et al., 2013; Li et al., 2020). Li et al. isolated a total of 13 cultivable bacterial strains from volcanic soil samples taken in the Turpan Basin (Li et al., 2020). Zhang et al. constructed a metagenomic library from soil samples of the Turpan Basin, and screened a novel β-galactosidase from an unculturable microorganism, which exhibited high thermostability and tolerance to reaction products (Zhang et al., 2013). Therefore, the investigation of prokaryotic community diversity in the hyper-arid environment is important not only for predicting the responses of ecosystems to environmental changes and enhancing adaptability across various ecological systems, but also for facilitating the biotechnological application of soil bacteria thriving in challenging and life-limiting conditions.

Although there is currently a significant amount of research focused on arid regions, studies on the biodiversity of hyper-arid areas in Xinjiang remain relatively scarce because the abundance of microbes in such environments can be very low, resulting in a narrow niche and low resource competitiveness (Feng et al., 2020). Thus, it is difficult to isolate microbes from extreme environments using traditional pure culture methods in the laboratory (Li et al., 2020). With the continuous development of sequencing technology in recent years, the research on extreme soil microbial ecology has made significant advances (Shu and Huang, 2022), among which the microbial community structure and functional regulation have become research hotspots (Shu and Huang, 2022; Viruel et al., 2022). Modern high-throughput sequencing technologies classified as next generation sequencing (NGS) can not only detect very small amounts of microbial DNA, but also deliver results that are closer to the real community structure of microorganisms due to the large sequencing volume and high-throughput of these methods. Hwang et al. investigated viral genomes from the Atacama Desert using Illumina HiSeq 2500 to reveal the diversity and ecological impact of viruses inhabiting hyper-arid soils (Hwang et al., 2021). Using the same sequencing platform, Le et al. compared the metagenomes of soils from extreme hyper-arid deserts to understand the relation between prokaryotic communities and stress responses in soil systems (Le et al., 2016). Although NGS machines based on Illumina sequencing have dominated the sequencing market, it has been reported that Illumina index hopping can introduce false-positive contamination, causing interference in studies on community composition, diversity, and community assembly mechanisms, particularly when studying microorganisms with low abundance. Recently, MGI Tech has introduced a series of new sequencers, including the DNA nanoball (DNB) sequencing platform (DNBSEQ-G400), which promises to provide high-quality sequencing data faster and at lower prices than Illumina sequencers (Jia et al., 2022).

Here, we decided to compare the performance of Illumina sequencing (NovaSeq 6000) with DNBSEQ-G400 for deep sequencing of prokaryotic 16S rRNA genes from hyper-arid Gobi soils in the Turpan Basin. Based on the results, we evaluated the impact of the sequencing platform on community analysis in extremely low biomass environments, and revealed the predominant prokaryotic community composition, diversity, and functions. We hope this work can provide guidance for the choice of sequencing platform, so as to better inform the sustainable development and reconstruction of fragile ecosystems in hyper-arid areas.

## Materials and methods

### Sampling sites

**Figure 1:**
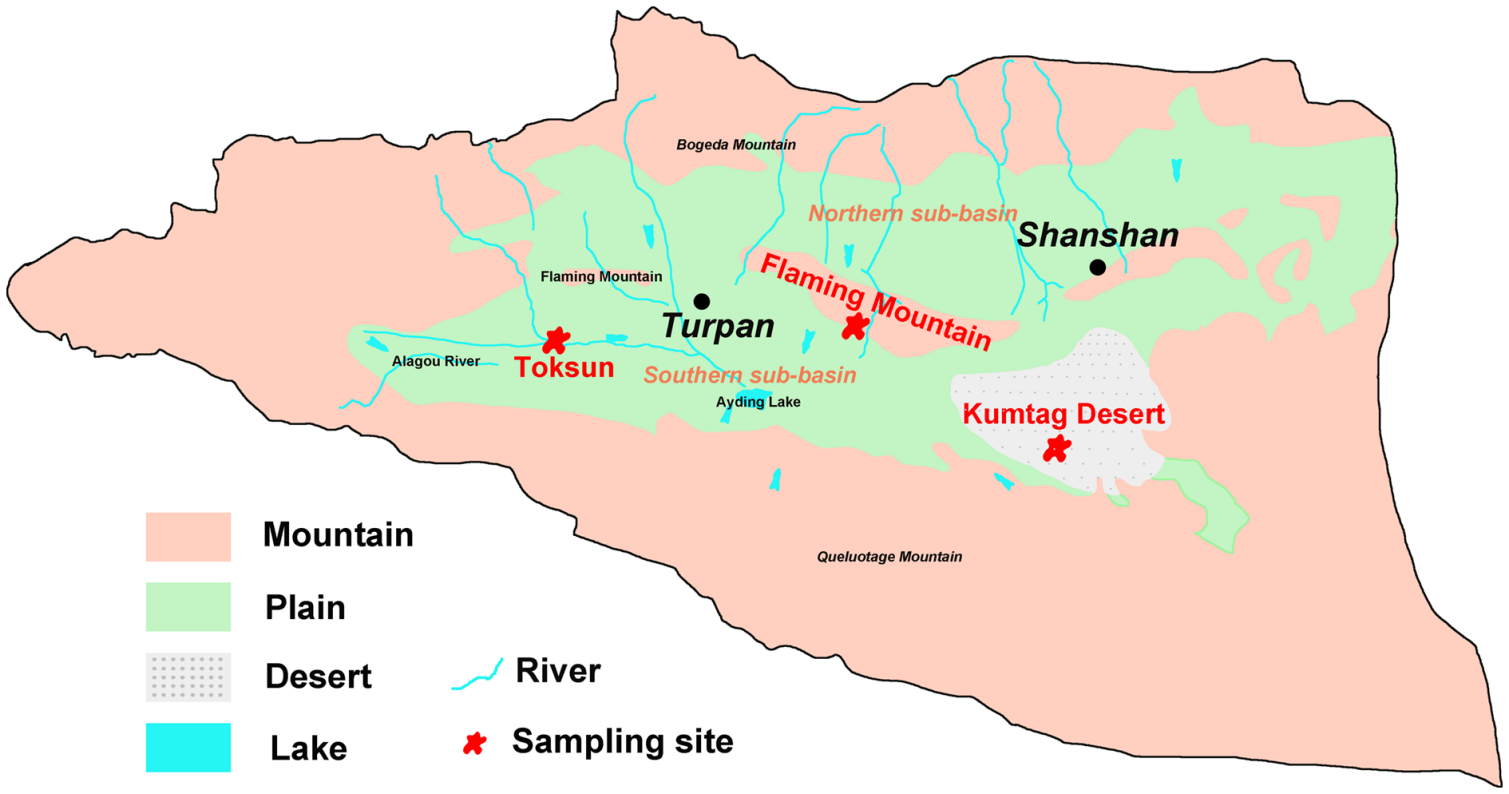
Hydrogeological map and sampling sites of Turpan Basin.

Field sampling was undertaken in June 2020 in the Turpan Basin, which is located at the middle east of Xinjiang Uyghur Autonomous Region of China (42°30′-43°20’ N, 87°50′-91°10′E). The Gobi Desert soil samples were collected at three sites (Figure 1), Toksun Desert (TKS), Flaming Mountain (FM), and Kumtag Desert (KMTG). The topography of this region is characterized by interlaced hills and plains with elevations ranging from −155 to 3,600 m (Pei et al., 2015). As a consequence of its extremely continental warm temperate location, Turpan has a climate with particularly high temperature, large temperature differences between day and night, large amounts of sunshine, strong solar irradiation, scarce rainfall, as well as frequent and strong winds (Pei et al., 2015). Therefore, this region experiences a hyper-arid climate with a mean annual precipitation of 6.9–25 mm, an average evaporation capacity of 2,727–3837.8 mm, and a mean annual temperature of 13°C, while the maximum temperature is over 49.6°C (Pei et al., 2015; Eminniyaz et al., 2017). The soil matrix in this region is mainly classified as sandy and gravelly, leading to the severe scarcity of vegetation and microorganisms in Gobi soil (Liu C. et al., 2021; Liu J. et al., 2021).

### Collection of soil samples

Eight samples were collected approximately 1 km apart in a circle with a diameter of 3 km at each sampling location (TKS, FM, and KMTG). At each sampling site, three samples were taken randomly from a 5 × 5 m homogeneous area and thoroughly mixed in sterile polypropylene bags after removing impurities. Finally, a total of 24 samples from three sampling locations were collected. All samples were obtained from a depth of 10–15 cm beneath the surface of the ground using a shovel. Finally, 2 kg of soil samples were obtained and stored at 4°C for soil physicochemical analyses and microbial quantification.

### Soil physicochemical analysis

Soil samples from each site were taken to the laboratory in Xinjiang Key Laboratory of Special Environmental Microbiology to conduct physicochemical and microbiological analyses. The soil water content was measured via the oven drying method. The pH of the soil was measured using a conventional pH meter by suspending the soil in distilled water at a ratio of 1:2.5 (w/v). The number of microbial colony forming units (CFU/g) was obtained using the serial dilution method and the spread plate counting method (He et al., 2023). Other indexes of soil samples were determined according to standard agrochemical analysis methods, including organic matter, total nitrogen, available nitrogen, available potassium, and soluble salt (He et al., 2023).

### DNA extraction and 16S rDNA amplicon sequencing

The genomic DNA was extracted from soil samples using a MP FastDNA 50 mL Spin Kit for Soil (MP Biomedicals, Santa Ana, CA, United States) according to the manufacturer’s instructions. In order to obtain as much DNA as possible, 200 g soil samples were partitioned into 20 portions of 10 g each and DNA was extracted in parallel. All extracted DNA were combined and washed with 1 mL of elution buffer. Finally, the purity and concentration of the extracted DNA were determined by agarose gel electrophoresis. An aliquot of extracted DNA was used as a template for amplification. The V4 region of the prokaryotic 16S rRNA gene was amplified using the universal primer pair 515F (5′-GTGCCAGCMGCCGCGGTAA-3′) and 806R (5′-GGACTACHVGGGTWTCTAAT-3′) (Jia et al., 2022).

For DNBSEQ-G400 amplicon sequencing library construction, a two-step PCR procedure was used as described previously (Jia et al., 2022). Briefly, the first-step PCR was conducted by inserting zero to three random nucleotides before each primer pair to ensure balanced nucleotide proportion at each position, thereby improving the accuracy of base-calling. The PCR temperature program encompassed an initial denaturation step at 95°C for 10 min, followed by 20 cycles of denaturation at 98°C for 20 s, annealing at 58°C for 30 s, and elongation at 72°C for 30 s, with a final elongation step at 72°C for 10 min. For the second PCR amplification, the primer with a sample barcode and the DNBSEQ sequencer adapter were used with a temperature program encompassing initial denaturation at 95°C for 5 min, followed by 15 cycles of denaturation at 98°C for 20 s, annealing at 58°C for 30 s, and elongation at 72°C for 30 s, with a final elongation step at 72°C for 10 min. After that, the PCR products were verified and purified by agarose gel electrophoresis. Finally, DNA nanoballs were constructed for sequencing on the paired-end 200-bp DNBSEQ-G400 platform (BGI-Qingdao). To construct libraries for Illumina NovaSeq 6000 amplicon sequencing, only a one-step PCR procedure was carried out using an initial denaturation step at 98°C for 1 min, followed by 30 cycles of denaturation at 98°C for 10 s, annealing at 50°C for 30 s, and elongation at 72°C for 30 s, with a final elongation step at 72°C for 5 min. After the purification of PCR products, the Illumina TruSeq DNA PCR-Free Library Preparation Kit (Illumina, United States) was used to construct sequencing libraries according to the manufacture’s protocol. Finally, the qualified libraries were sequenced on the paired-end 250-bp Illumina NovaSeq 6000 platform at Novogene Co., Ltd. (Beijing, China).

### Bioinformatic analysis

The paired-end (PE) reads generated by the high-throughput sequencing platforms were assigned to samples based on their unique barcodes and truncated by cutting off the barcode and primer sequence. The raw reads were filtered using SOAPnuke (v1.5.6) to remove adapter sequences and low-quality reads. Then, these high-quality clean reads were assembled into clean tags using FLASH (v1.2.11). Reads generated by each of the two sequencing platforms were combined and the denoising clustering algorithm unoise3 was applied to produce zero-radius operational taxonomic units (ZOTUs) in USEARCH (v10.0.240). The ZOTU taxonomic assignment at different taxonomic levels (from phylum to genus) was analyzed using the RDP training set (v18) with a 0.8 confidence cutoff value. QIIME (v1.9.1) was used to evaluate α-diversity (Observed_species, Simpson and Chao 1 indices) as well as the weighted and unweighted UniFrac and Bray-Curtis β-diversity distances (Liu et al., 2023), and rarefaction curves of observed species were drawn by the function “plot” of R.

### Statistical analysis

All statistical analyses were performed using R software (v3.4.1). The significance of differences in α-diversity or phylogenetic diversity was assessed using the Wilcoxon-test. Differences of weighted and unweighted UniFrac distances were assessed using PERMANOVA in the “vegan” R package. The differences in community structure of the different samples and groups were analyzed using principal coordinate analysis (PCoA) based on weighted and unweighted calculations. The Sloan neutral community model prediction was carried out using the “MicEco” package in R. The beta Nearest Taxon Index (βNTI) values were calculated using the “picante” package in R. The co-occurrence networks of the prokaryotes were constructed using the SparCC algorithm and visualized by “igraph” package in R. PICRUST (v1.1.1) was used for predicting the functions from the 16S rRNA gene sequences according to the taxonomy affiliations.

## Results

### Soil physicochemical characteristics

The soil physicochemical characteristics were measured at three different hyper-arid Gobi sampling sites in Turpan Basin (TKS, FM and KMTG). The geochemical parameters were recorded for 8 soil samples at each site, including organic matter (OM), total nitrogen (N), available nitrogen (IonN), available potassium (IonK), soluble salt (Ss), soil water (Sw), and pH. As shown in Table 1, all samples were slightly alkaline, with pH values ranging from 8.42 ± 0.13 to 8.90 ± 0.14, and had extremely low moisture content (0.189 ± 0.133 ~ 0.251 ± 0.044%), which was consistent with other dry deserts (Liu L. et al., 2022; Liu S. et al., 2022). The concentration of OM was higher at FM (7.875 ± 3.029 g/kg) than at KMTG (3.188 ± 1.208 g/kg) and TKS (3.625 ± 1.370 g/kg), resulting in a high microbial count at FM (198 ± 37 CFU), while a low microbial counts at KMTG (59 ± 24 CFU) and TKS (67 ± 9 CFU). Furthermore, the content of N, IonK, and Ss were also significantly higher at FM than at KMTG and TKS. However, the soil characteristics did not vary significantly between KMTG and TKS, with the exception of IonN and Ss.

**Table 1:** Different physicochemical parameters and microbial count (CFU/g) of hyper-arid Gobi soils in FM, KMTG, and TKS.

Physicochemical parameters	FM	KMTG	TKS
Organic matter (OM, g/kg)	7.875 ± 3.029	3.188 ± 1.208	3.625 ± 1.370
Total nitrogen (N, g/kg)	0.18 ± 0.067	0.091 ± 0.075	0.068 ± 0.073
Available nitrogen (IonN, mg/kg)	8.738 ± 5.619	0.638 ± 0.819	17.05 ± 16.892
Available potassium (IonK, mg/kg)	275.825 ± 112.216	151.963 ± 50.153	155.362 ± 50.146
soluble salt (Ss, g/kg)	420.3 ± 181.056	125.313 ± 107.243	33.65 ± 30.065
pH	8.90 ± 0.14	8.54 ± 0.21	8.42 ± 0.13
soil water (Sw, %)	0.251 ± 0.044	0.189 ± 0.113	0.229 ± 0.102
Microbial count (CFU/g)	198 ± 37	59 ± 24	67 ± 9

### Overview of sequencing data

Two typical NGS technologies were used to sequence the 16S rRNA genes of microorganisms isolated from this hyper-arid environment. The sequencing statistics were summarized in Supplementary Table S1. A total of 2,176,485 and 3,088,056 PE reads were generated from 24 examined samples (8 samples per site) using Illumina NovaSeq 6000 and DNBSEQ-G400, respectively. After PE assembly and filtering from raw tags, 2,046,090 (NovaSeq 6000) and 2,275,748 (DNBSEQ-G400) clean tags with high-quality were obtained, from which 1,560,103 (NovaSeq 6000) and 2,146,520 (DNBSEQ-G400) effective tags were further produced by removing the chimeric sequences from clean tags, accounting for 71.7% (NovaSeq 6000) and 69.5% (DNBSEQ-G400) of the total quantified sequences. The average effective tags length across different samples was 253 bp, and Q20 was more than 96.61%. After the evaluation of sequencing data quality based on GC content and Q30, we concluded that all of the parameters met the demands for further analysis.

### Comparison between the MGI and Illumina sequencing platforms

**Figure 2:**
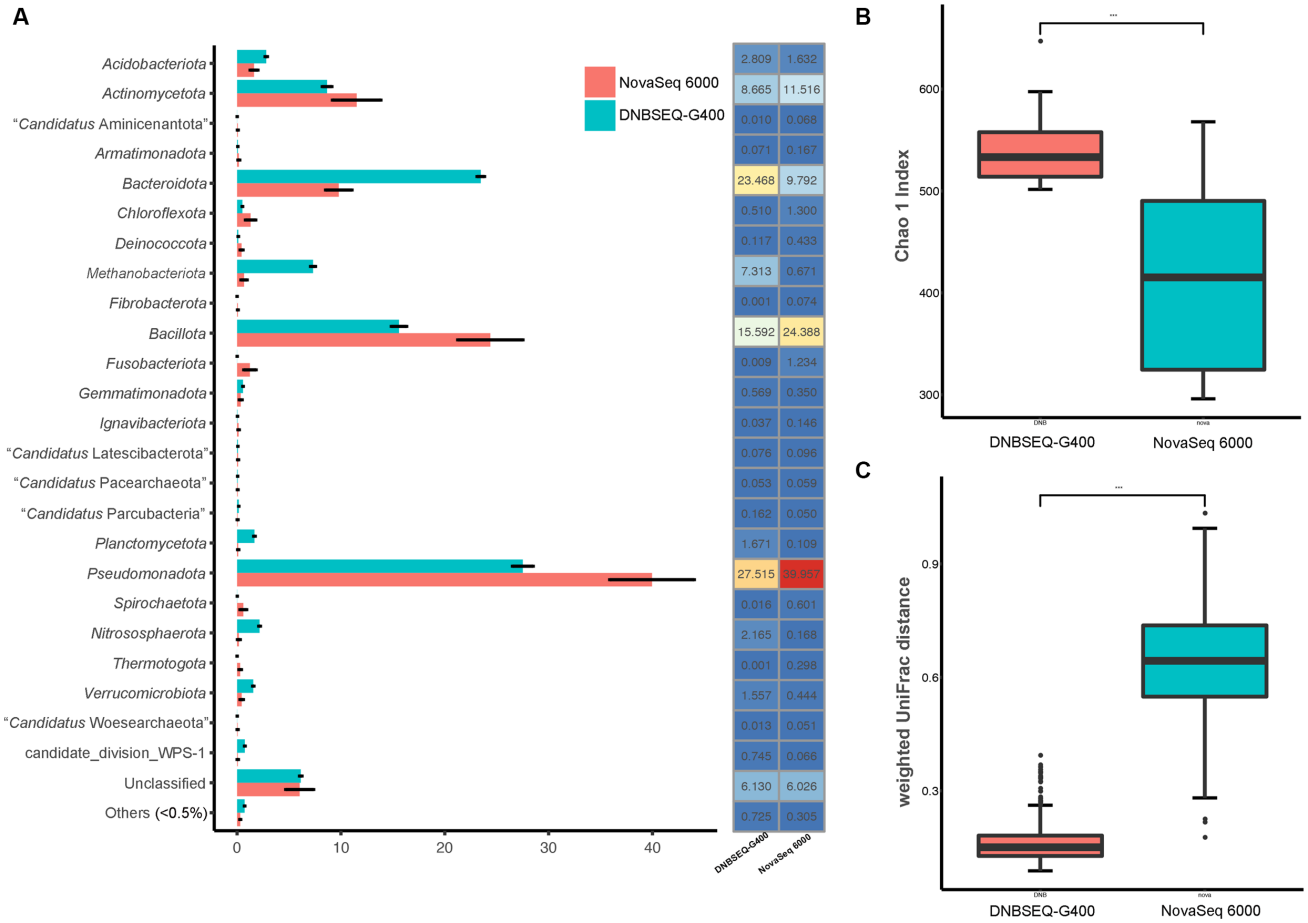
**(A)** Relative abundance at phyla level, **(B)** α-diversity (Chao 1 index) and **(C)** β-diversity index (weighted UniFrac distance) of prokaryotic community in Turpan Basin identified by NovaSeq 6000 and DNBSEQ-G400 platforms (***p < 0.001).

In order to more accurately reflect the composition of prokaryotic communities in the low-biomass hyper-arid environment, the prokaryotic species diversity was assessed using both Illumina NovaSeq 6000 and DNBSEQ-G400. According to the analysis of the high-throughput sequencing results, the samples contained a total of 36 phyla (Figure 2A). The dominant phyla were *Pseudomonadota*, *Bacillota*, *Bacteroidota*, and *Actinomycetota*, with an average relative abundance of 39.96, 25.39, 9.79, and 11.52% according to NovaSeq 6000, as well as 27.52, 15.59, 23.47, and 8.67% according to DNBSEQ-G400. However, 4 phyla (*Elusimicrobiota*, *Lentisphaerota*, “*Candidatus* Microgenomatota,” “*Candidatus* Poribacteriota”) were only identified by DNBSEQ-G400, and 3 phyla (“*Candidatus* Acetothermia,” “*Candidatus* Cloacimonadota,” *Deferribacterota*) were only identified by NovaSeq 6000, with low abundance (<0.5%). Although the identification at the phylum level was relatively consistent between NovaSeq 6000 and DNBSEQ-G400, the former platform exhibited marginally larger error bars, indicating the poor repeatability. In addition, the Chao1 index, one of the indices used to evaluate microbial α-diversity in ecology, was much higher according to the DNBSEQ-G400 platform than according to the NovaSeq 6000 (Figure 2B), indicating that a larger proportion of prokaryotic diversity was identified by DNBSEQ-G400. Similarly, β-diversity (weighted UniFrac distance) result showed that the DNBSEQ-G400 platform presented good repeatability with relatively close distances between samples (Figure 2C).

**Figure 3:**
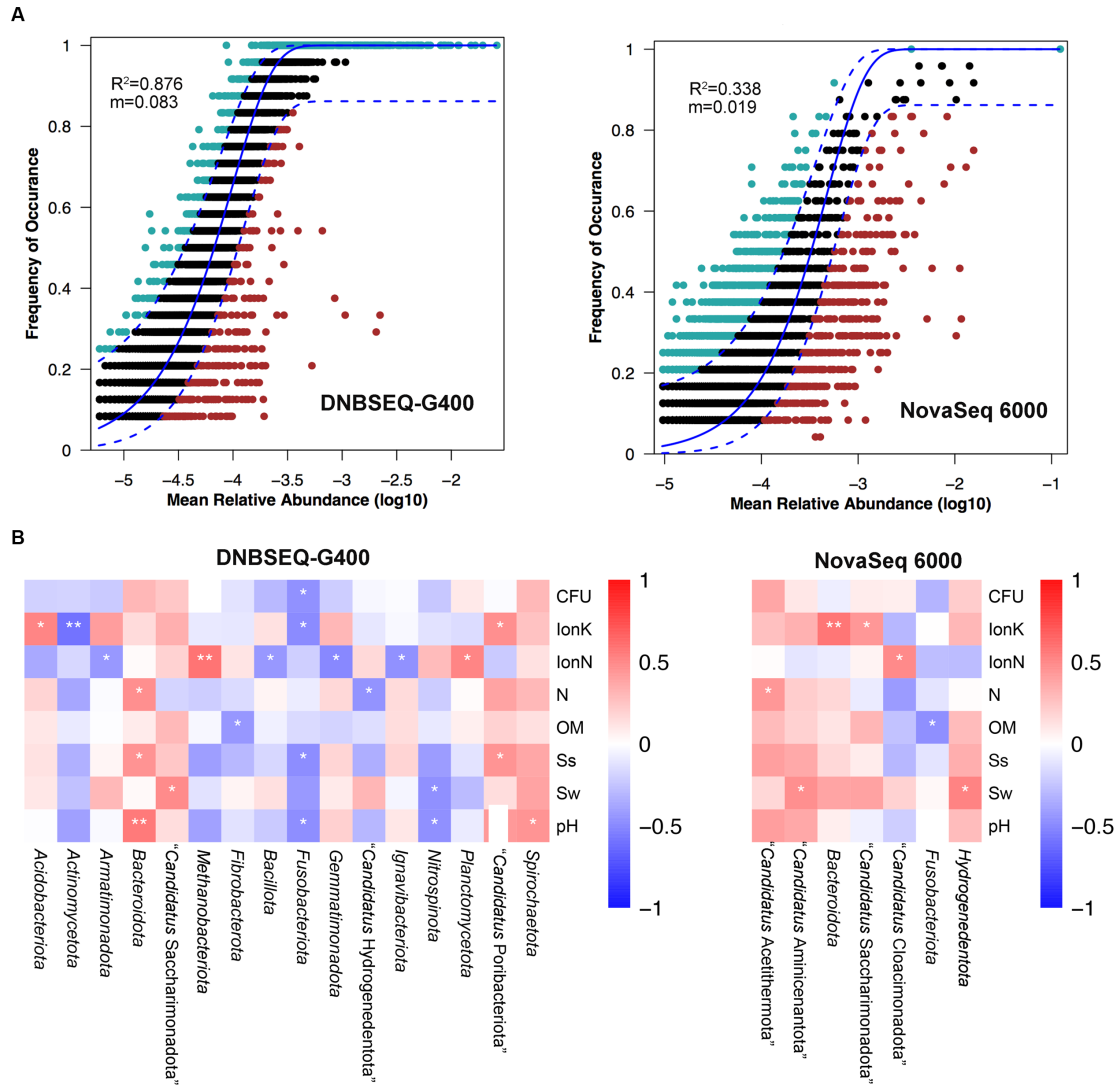
**(A)** Fit of Sloan’s neutral model for analysis of community assembly processes. **(B)** The relationship between top phylum and environment factors of Spearman heatmap correlation analysis (**p ≤ 0.01, *0.01 < *p* ≤ 0.05).

Next, a potential niche-neutrality balancing model was used to compare the internal assembly mechanism of the prokaryotic community and its abundance distributions (Wang L. et al., 2021; Wang X. et al., 2021). The Sloan neutral model was used to investigate the microbial community assembly process. There are two important and complementary types of processes controlling the assembly of microbial communities, deterministic and stochastic process (Wang L. et al., 2021; Wang X. et al., 2021; Jia et al., 2022). As shown in Figure 3A, the Sloan neutral model-based analysis revealed that community assemblages of the DNBSEQ-G400 platform were well described by neutral-based models, with a relatively high coefficient fit (*R*^2^ = 0.876), which indicated that the prokaryotic community assembly process was primarily mediated by stochastic process (Jia et al., 2022). By contrast, the fitting result of NovaSeq 6000 platform showed a relatively weak coefficient (*R*^2^ = 0.338). Jiao et al. reported that the deterministic assembly was dominant in microbial communities in agricultural, forest, and grassland soils, whereas stochastic assembly contributed a larger fraction to the assembly of microbial communities in desert soils (Jiao et al., 2022). In addition, we also found that more prokaryotic phyla identified by DNBSEQ-G400 were significantly correlated with environmental factors (Figure 3B). In particular, *Actinomycetota* and *Fusobacteriota* presented negative correlation with all environmental factors, while *Bacteroidota* and *Spirochaetota* were positively correlated with environmental factors. It has been reported that *Actinomycetota* and *Bacillota* are the dominant phyla in arid soils worldwide, as they are well adapted and can survive in such barren soil with drought and high salt content (Gao et al., 2019).

In conclusion, the comparison between the NovaSeq 6000 and DNBSEQ-G400 sequencing platforms finally verified that the latter offered high accuracy for the identification of community diversity, whereby its results better matched the actual environmental conditions for the prokaryotic assembly mechanism. Therefore, we further investigated the predominant prokaryotic community composition, diversity, and prokaryotic functions at three typical hyper-arid sites using only the DNBSEQ-G400 sequencing results in the following study.

### Diversity analysis using the DNBSEQ-G400 sequencing platform

After clustered into ZOTUs at 100% identity thresholds, the clean tags sequenced by DNBSEQ-G400 platform were clustered into 13,161 ZOTUs. Among them, 447 ZOTUs were unique to TKS, 668 to FM, and 445 to KMTG (Supplementary Figure S1), indicating a negligible difference of prokaryotic ZOTU numbers across the soil samples. Moreover, 51.8% of prokaryotic ZOTUs were shared by all samples. The rarefaction curves of all samples tended to approach saturation plateau (Supplementary Figure S2), indicating that the sequencing depth could reasonably explain the diversity of prokaryotic communities (Liang et al., 2020). As mentioned above, a total of 36 prokaryotic phyla were detected across all soil samples. Dominant phyla were *Pseudomonadota*, *Bacteroidota*, *Bacillota*, *Actinomycetota*, *Methanobacteriota*, *Acidobacteriota*, *Nitrososphaerota*, and *Planctomycetota* (Supplementary Figure S3). In addition, the unclassified prokaryotic communities accounted for an average of 8% of the total relative abundance at the phylum level.

**Figure 4:**
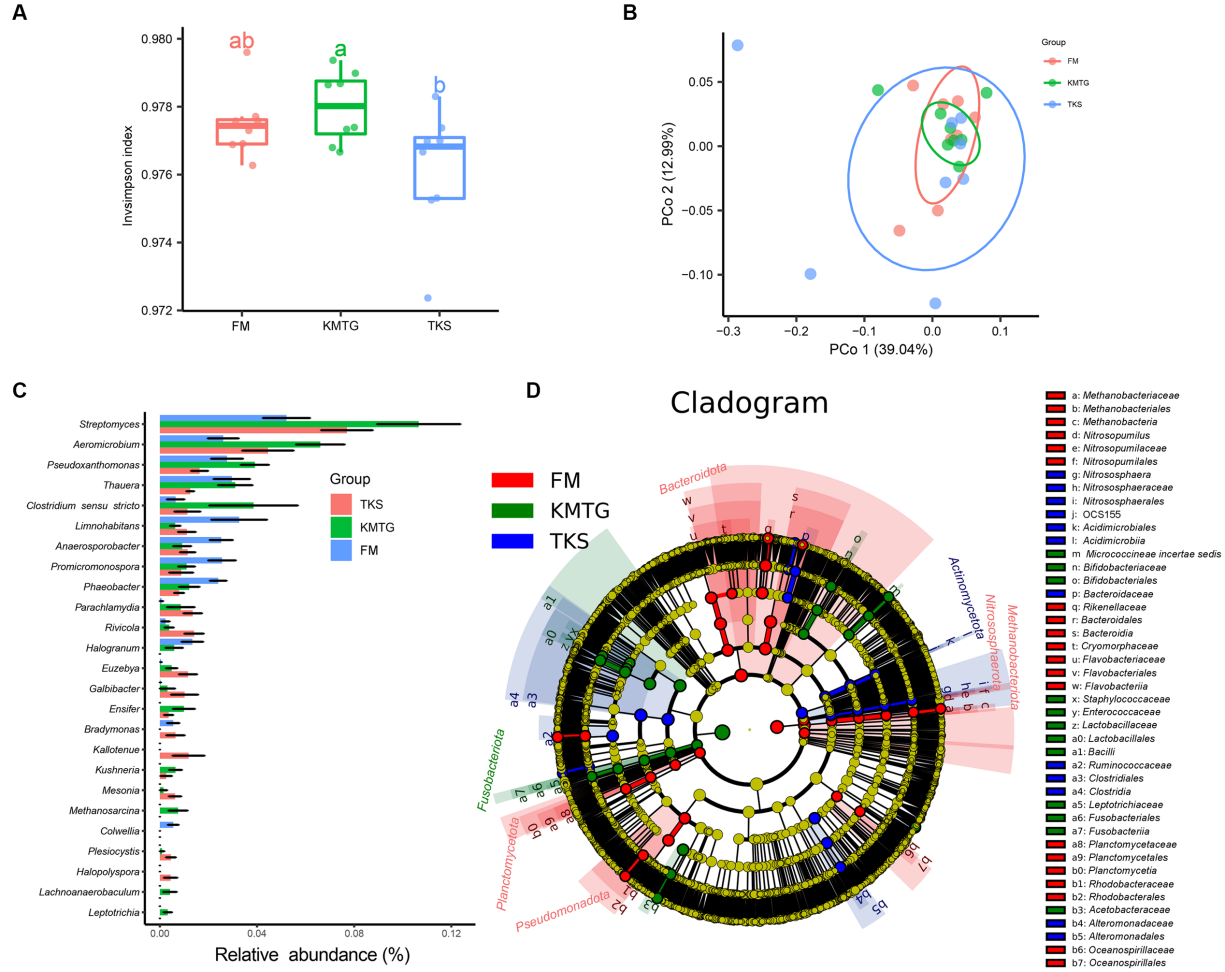
α- and β-diversity of prokaryotic communities at FM, KMTG, and TKS reflected by **(A)** Invsimpson indices and **(B)** Principal co-ordinate analysis (PCoA) (PERMANOVA test: 999 permutations, *p* = 0.375). **(C)** The 20 genera with significant difference by Kruskal-Wallis test. **(D)** LEfSe analysis of soil prokaryotic abundance at six-level cladogram (from kingdom to genus).

The calculated α-diversity indices were used to evaluate the diversity of the prokaryotic communities. The Invsimpson indices of all samples displayed a high value (>0.974) across the three regions, indicating a great diversity of prokaryotic communities (Figure 4A). No statistically significantly differences in α-diversity were found. However, the diversity of samples from FM was relatively concentrated, while it was more discrete for KMTG and TKS. Principal coordinates analysis (PCoA) was performed to evaluate the prokaryotic community composition based on Bray–Curtis dissimilarity index. In the hyper-arid soil, PCoA1 (39.04%) and PCoA2 (12.99%) explained 52.03% of the total microbial variation (Figure 4B). Prokaryotic community from three sites grouped together with close Bray-Curtis distance, indicating that there were some similarities between all sampling sites. The results also showed that the repeatability of samples from FM was better than that for KMTG and TKS, which was relatively discrete.

To further study the prokaryotic community structure, the differences of prokaryotic community composition in the three regions was analyzed at the taxonomy level. The Kruskal-Wallis test was first used to compare the relative abundance at the genus level among the three sites. As shown in Figure 4C, 20 genera with significant differences between FM, KMTG and TKS were identified. The top 5 prokaryotic genera from KMTG, i.e., *Streptomyces*, *Aeromicrobium*, *Pseudoxanthomonas*, *Thauera*, and *Clostridium sensu stricto* had a significantly higher abundance than at TKS and FM. However, the relative abundance of *Limnohabitans*, *Anaerosporobacter*, *Promicromonospora*, and *Phaeobacter* was higher at FM than at KMTG and TKS. Then, LEfSe analysis (linear discriminant analysis effect size; LDA cutoff ≥3) was used to illustrate the taxonomic differences from phylum to genus. In Figure 4D, each circle at a different classification level in the evolution map represents a classification at that level. The results showed that different groups at different taxonomic levels could be distinguished across sites. A total of 44 biomarkers were detected in the three regions, whereby more prokaryotic taxa were detected at FM (20 biomarkers) than at KMTG (12 biomarkers) and TKS (12 biomarkers), namely *Bacteroidota* (7 biomarkers from class to family), *Planctomycetota* (3 biomarkers from order to family), *Nitrosophaerota* (3 biomarkers from order to genus), *Methanobacteriota* (3 biomarkers from class to order), and *Pseudomonadota* (4 biomarkers from order to family). *Fusobacteriota* and *Actinomycetota* acted as a leading discriminant clade at KMTG and TKS, respectively.

### Co-occurrence network patterns of prokaryotic communities affected by environmental factors

**Figure 5:**
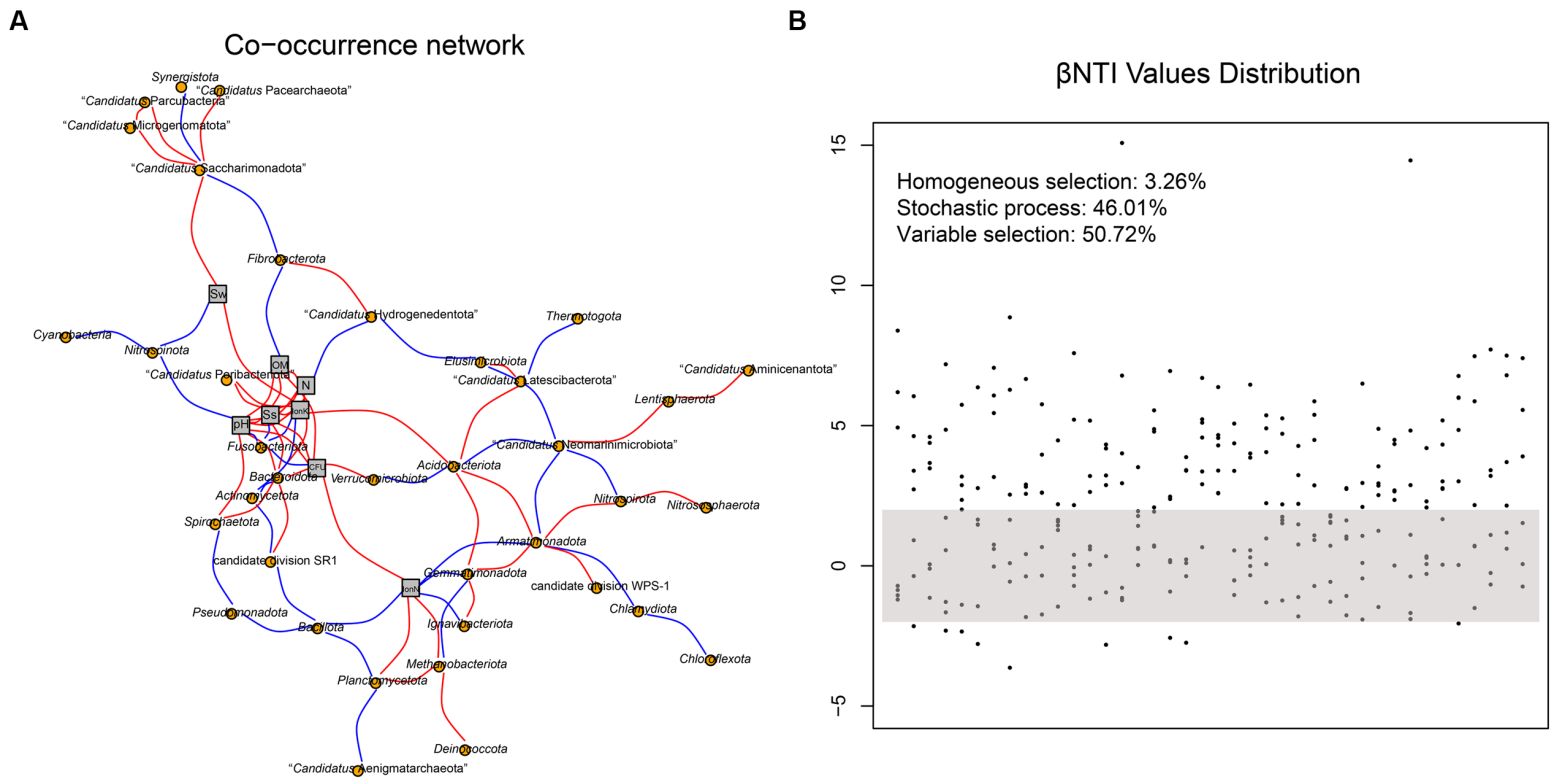
**(A)** Co-occurrence network based on correlation analysis between species at phylum level and environmental factors. Sw, Soil water content; OM, Organic Matter; N, Total Nitrogen; Ss, Soil salt; IonK, available potassium; IonN, available Nitrogen. **(B)** The distribution of βNTI values. Each point represents a βNTI value. A |βNTI| value of less than 2 (gray shaded region) indicates stochastic assembly processes; a βNTI value of less than −2 indicates a homogeneous selection; and a βNTI value of greater than 2 indicates a variable selection.

The co-occurrence network of soil prokaryotic communities was constructed to explore the correlation of core microbial taxa and environmental factors. The correlation was statistically significant, and the soil prokaryotic network consist of 37 nodes (phylum) and 87 edges. As shown in Figure 5A, generally, the soil microbes in this co-occurrence group showed different correlations with different environmental factors. For example, *Nitrospinota* and *Fusobacteriota* exhibited negative correlations with pH, while *Spirochaetota* showed a positive correlation, which was consistent with the results shown in Figure 3A. The abundance of the majority of microorganisms was positively correlated with Ss, IonK, N, OM, and CFU, resulting in a more complex network. Such strong correlations with these environmental factors indicated that they have an important role in the composition of the prokaryotic community in hyper-arid soil. We then calculated the β-nearest taxon index (βNTI) to evaluate the changes in the relative influences of deterministic and stochastic assembly processes in hyper-arid soil. As shown in Figure 5B, the fractions of community assembly process explained by homogeneous selection (βNTI≤ − 2), stochastic process (|βNTI| < 2), and variable selection (βNTI≥2) were determined (Jia et al., 2022). The distribution of βNTI between −2 and 2 accounted for 46.01%, indicating that prokaryotic community assembly in hyper-arid area was driven by stochastic process, which was consistent with the results of Sloan’s neutral model. In addition, variable selection (50.72%) also greatly influenced the prokaryotic community assembly, demonstrating that community composition was susceptible to environmental microbial migration in hyper-arid soil.

### Prokaryotic functional predictive analysis

**Figure 6:**
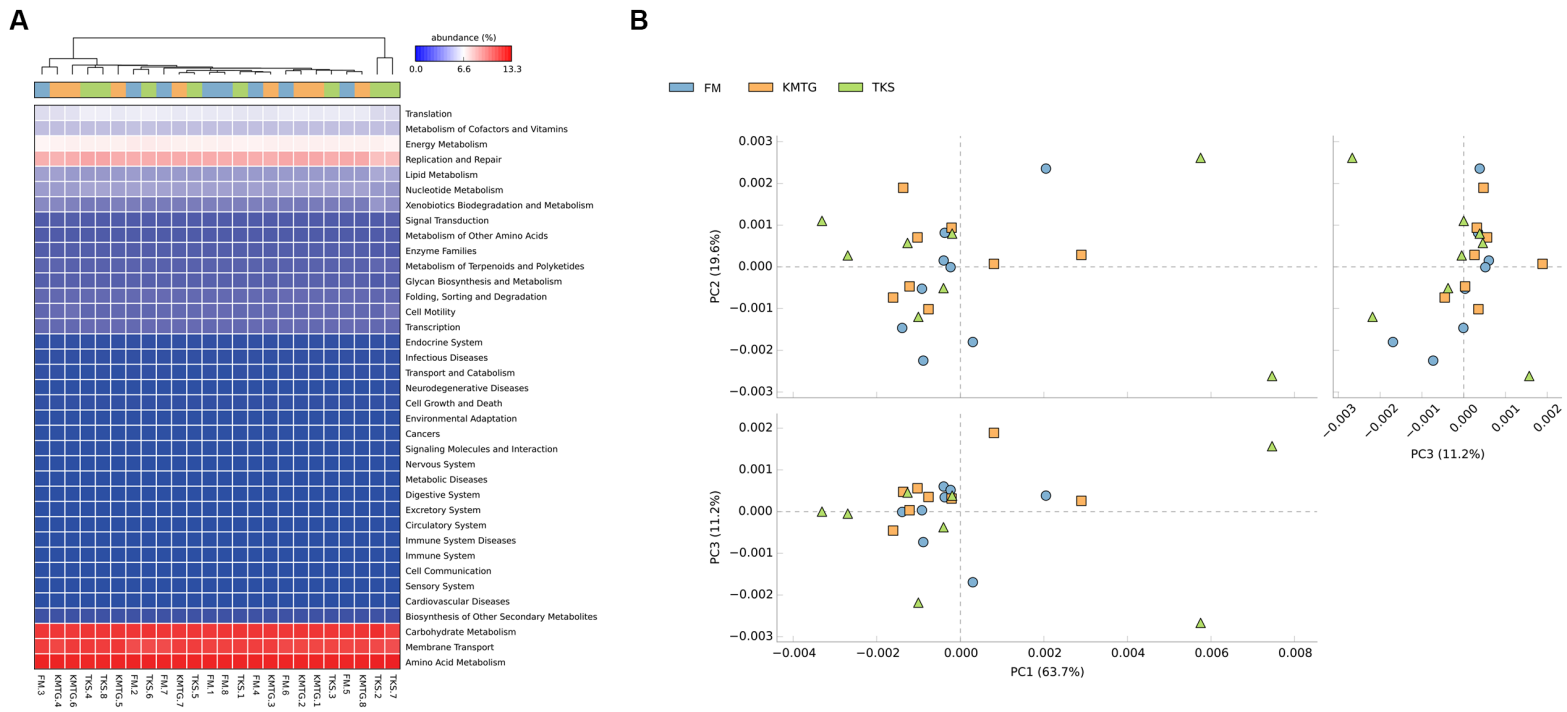
**(A)** Heatmap of the relative abundances of PICRUSt predicted genes for different samples. **(B)** Principal Component Analysis (PCA) of metabolic pathways (ANOSIM test: 999 permutations, *R* = 0.045, *p* = 0.375).

PICRUSt software was employed to predict gene functions of soil microbiota identified in hyper-arid soil based on the annotations in the Kyoto Encyclopedia of Genes and Genomes (KEGG) database (Wu et al., 2021). According to the prediction results (Figure 6A), 24 samples collected from three regions were mainly enriched in 37 metabolic pathways. The heatmap of metabolic pathways showed that the relative abundance of functions was generally similar among the samples. Among them, the majority of predicted sequences were associated with prokaryotic functions involved in carbohydrate metabolism, membrane transport, amino acid metabolism, replication and repair, as well as energy metabolism, indicating that the soil microorganisms in the Turban Basin had a particularly high utilization of carbon sources (Liu L. et al., 2022; Liu S. et al., 2022). The metabolic pathways with the most significant differences were biotin metabolism, CAM ligands, cytosolic DNA-sensing pathway, ECM-receptor interaction, focal adhesion, and primary bile acid biosynthesis (Supplementary Figure S4). In addition, a Principal Component Analysis (PCA) based on KEGG database was used to compare the global metabolic changes of the microbiota. The results showed that the three groups were not significantly different in the functional pathways (Figure 6B), illustrating that the soil microbial communities from different hyper-arid regions of the Turpan Basin were functionally convergent.

### The special prokaryotic populations analysis

**Figure 7:**
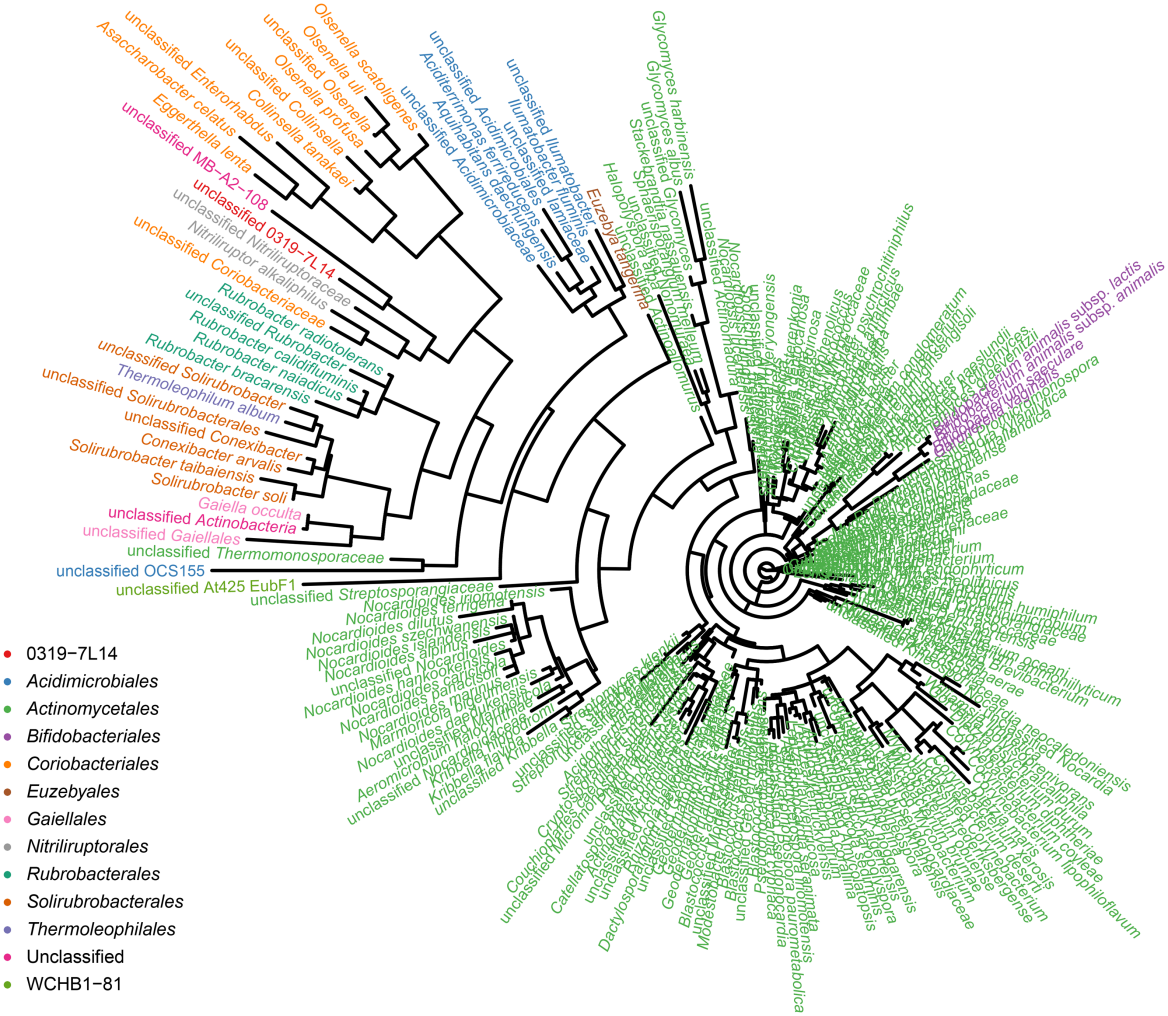
Phylogenetic tree for *Actinomycetota* species.

To identify the prokaryotic populations in this hyper-arid region, the relative abundance of taxa at the phylum, class, order, family, and genus levels of prokaryotic communities was analyzed. As elaborated in Supplementary Figure S5, the top 6 phyla were *Pseudomonadota*, *Bacteroidota*, *Bacillota*, *Actinomycetota*, *Methanobacteriota*, and *Acidobacteriota*. However, more than 50% of the 16S rRNA sequences could not be classified into genera and species, indicating that large numbers of rare or unknown genera and species were existed in this hyper-arid region. In our previous work, we found that *Actinomycetota* were the major phyla at FM (He et al., 2023), therefore the diversity of *Actinomycetota* were further discussed here. The phylogenetic analysis showed that the microorganisms of the Turpan Basin represented a considerable level of taxonomic diversity (Figure 7). There were 247 *Actinomycetota* belonging to 12 orders, including 0319-7 L14, *Acidimicrobiales*, *Actinomycetales*, *Bifidobacteriales*, *Coriobacteriales*, *Euzebyales*, *Gaiellales*, *Nitriliruptorales*, *Rubrobacterales*, *Solirubrobacterales*, *Thermoleophilales*, and WCHB1-81. At the lower taxonomic levels, there were 86 families, 214 genera, and 247 species, demonstrating the diversity of *Actinomycetota* in this hyper-arid environment.

## Discussion

The climate of the Turpan Basin is extremely dry and hot, especially in Toksun County, where the highest recorded temperature is 49.6°C, and the average annual precipitation is only 6.9 mm, making it the most arid place in China (Eminniyaz et al., 2017). It is precisely due to the extreme climate that the unique ecological environment in Turpan area has been formed, also including FM and KMTG, which constitutes a good model system to investigate the impact of hyper-arid conditions on microbial assemblages in the soil. All regolith soils in this area are mainly composed of sandy with saline-alkaline soils (Liu C. et al., 2021; Liu J. et al., 2021). The physicochemical parameters indicated that all samples in this study had very low contents of moisture, organic matter and total nitrogen, which was similar to previously described samples from the Qaidam Basin (Liu L. et al., 2022; Liu S. et al., 2022). By comparing the three regions, we found that the organic matter, total nitrogen, available potassium, and soluble salt in FM area had a relative high content than at KMTG and TKS, indicating that more nutrients were available for the growth of soil microorganisms. Consistently, the average microbial count in the FM region was more than three times that of the KMTG and TKS samples. In summary, the soil characteristics of different regions of the Turpan Basin exhibited obvious differences, leading to the spatial heterogeneity of this area.

The extreme habitat of the Turpan Basin is inhospitable to most forms of life, but it provides a haven for the thriving diversity of thermophiles (Parihar et al., 2022). However, information on the prevalence and complexity of these soil microbiota cannot be obtained by traditional cultivation methods because most microorganisms from the soil cannot be cultured in the laboratory (Li et al., 2020). In recently years, 16S rRNA amplicon sequencing technology has provided an effective strategy to analyze the microbial diversity of extreme environments (Parihar et al., 2022). Two major DNA sequencing platforms from two companies (Illumina Inc., NovaSeq 6000; MGI Tech Co., DNBSEQ-G400) were used to compare which was better able to reflect microbial diversity in hyper-arid soils with extremely low biomass abundance. The SOAPnuke results showed that both platforms provided high-quality sequencing data with sufficient coverage that were suitable for ZOTU analysis. A comparison of the two platforms revealed that the performance of DNBSEQ-G400 was mostly concordant with NovaSeq 6000, and might break the domination of Illumina in the sequencing market by offering lower prices (Anslan et al., 2021). Moreover, according to the analysis of the high-throughput sequencing results, more prokaryotic phyla could be identified based on DNBSEQ-G400 results. Some of these had notable features, such as *Elusimicrobiota*, “*Candidatus* Poribacteriota,” and *Lentisphaerota*, isolated from semi-arid savanna soil and hyper-arid intermontane basin in the Qaidam Basin (Cheng et al., 2016; Xing et al., 2019), which were capable of reducing nitrate or sulfur compounds. The relative abundance and diversity index obtained from the DNBSEQ-G400 platform resulted in a relatively low error range and a high value, indicating the better repeatability and high prokaryotic diversity. Notably, it was reported that 0.2 ~ 6% of index misassignment rate could occur on the Illumina sequencing platform, leading to the potential misinterpretation of sequencing results (Jia et al., 2022). Our previous work also observed a significantly lower fraction of potential false positive reads for DNBSEQ-G400 compared to NovaSeq 6000, indicating the superiority of the DNBSEQ-G400 sequencing platform (Jia et al., 2022).

The importance of disentangling community assembly mechanisms is widely recognized in microbial ecology, which is beneficial for better understanding the maintenance and generation of terrestrial microbial diversity (Zhang et al., 2019). The assembly of microbial communities is controlled by stochastic and deterministic processes, with each process governing differential fractions of microbial community compositions across diverse ecosystems. The Sloan’s neutral model analyzed for DNBSEQ-G400 revealed a prominent role of stochastic process in forming the prokaryotic community. It had been reported that soil biota tended to enter a dormant state to cope with hyper-arid environment, which contributes to the resistance to environmental stressors and consequently weakens deterministic processes (Kang et al., 2022). In addition, null model analyses based on phylogenetic turnover revealed that soil prokaryotic community from hyper-arid regions were subject to the combined effects of stochastic process (|βNTI| < 2) and variable selection (βNTI > 2). Our results revealed the prominent role of stochastic process in forming the prokaryotic community of the hyper-arid Turpan Basin. Meanwhile, the contribution of variable selection in regulating prokaryotic community structure should not be neglected, indicating that community composition is susceptible to the migration of environmental microbes.

Further, Spearman correlation analysis showed that more prokaryotic phyla identified by DNBSEQ-G400 were related to soil physicochemical parameters. It had been reported that pH was the key environmental factor affecting the distribution of prokaryotic community structure, which is directly or indirectly related to available nitrogen, phosphorus, organic carbon, and metal ions (Li et al., 2021). A previous study found that the relative abundance of *Bacteroidota* and *Acidobacteriota* in the desert soil of the Ebinur Lake Basin had a significantly correlation with the pH (Li et al., 2021). *Bacteroidota* and *Acidobacteriota* had a significant positive correlation and an extremely significantly negative correlation with pH, respectively. In our study, a similar result was also obtained for DNBSEQ-G400 sequencing platform. Moreover, the Sloan neutral model results indicated the general dominance of stochastic processes, which were reported to be more pronounced in drier soils, so that the phylogenetic structure of the community was more randomly assembled (Lee et al., 2018). All these results depicted that the prokaryotic community structure and assembly processes gleaned from the DNBSEQ-G400 data were more consistent with the characteristics of the real environment.

Considering the superiority of the DNBSEQ-G400 sequencing platform, the prokaryotic diversity was analyzed according to its sequencing results. In this study, we found that the three sampling areas had no significant differences in Simpson indices and PCoA, indicating that the prokaryotic diversity is generally similar in this area. The composition of soil microbes in the Turpan Basin arid desert was homogeneous, and dominated by xerotolerant, halotolerant, and radioresistant *Pseudomonadota*, *Bacteroidota*, *Bacillota*, *Actinomycetota*, *Methanobacteriota*, *Acidobacteriota*, *Nitrososphaerota*, and *Planctomycetota*. Among them, *Pseudomonadota* was the predominant prokaryotic phylum, which are abundant free-living bacteria in many oligotrophic habitats, such as the hyper-arid Atacama Desert of northern Chile, or the sandy subsurface soils of Virginia and Delaware (Cao et al., 2017). In addition, environmental variables showed a significant correlation with the prokaryotic community composition. A large number of studied showed that soil prokaryotic communities are highly sensitive to changes of pH, soil organic matter and the availability of soil mineral nutrients (Daniel et al., 2018; Shao et al., 2019; Liu C. et al., 2021; Liu J. et al., 2021). In this work, we found that several dominant environmental factors, including pH, soluble salt, organic matter, total nitrogen, and available potassium, exhibited a complex network with greater connectivity, which was considered more robust to environmental stresses than simple networks with less connectivity (Wang L. et al., 2021; Wang X. et al., 2021). In addition, these environmental factors could also impact the distribution of dominant prokaryotic phyla. Overall, the prokaryotic community was mainly influenced by positive correlations, indicating that available nutrients and minerals in the soil were the main limiting factors for prokaryotic colonization.

The microbiota of hyper-arid soil are important participants in biogeochemical cycles, and PICRUSt functional predictive analysis based on high-throughput sequencing has begun to be applied in the assessment of metabolic functions, which is beneficial for exploring the adaptation of microbial communities to different environmental conditions (Zhang Q. et al., 2021; Zhang Y. et al., 2021). This study found that there was a high diversity of prokaryotic functions at the three sampling sites, and PCA results indicated that there were no significant differences in the functional pathways, showing the functional convergence of prokaryotes in the Turpan Basin. Among them, amino acid metabolism, membrane transport, and carbohydrate metabolism were the main metabolic pathways at three regions, which was in line with the previous literature on arid land (Sun et al., 2020). Amino acid metabolism enables microbes to convert ammonium salts, nitrates, and other inorganic nitrogen absorbed from the environment into proteins. Carbohydrate metabolism is a vital biochemical process that regulates the formation, decomposition, and mutual transformation of carbohydrates in microbes. Finally, membrane transport can regulate the osmotic potential of microbial cell to adapt to drought stress (Xiao et al., 2021). The enrichment of the above three metabolic pathways affected the surrounding soil nutrients in hyper-arid areas, promoting the growth of vegetation, and thus creating biodiversity in arid desert environments.

Microbial resources in hyper-arid areas have attracted significant attention from microbiologists, as water availability is a major limiting factor for all forms of life. Although low moisture and water, extreme temperature, and poor nutrients limit the growth of microorganisms, some extremophiles could still adapt to live under these harsh conditions. *Actinomycetota* are among the most frequent groups, generally accounting for over 35% of all microorganisms in hyper-arid areas (He et al., 2023). It has been reported that the *Actinomycetota* communities plays an important role in the ecosystems they colonize, as they are capable of fixing CO_2_ via the Calvin-Benson-Bassham cycle to supply organic carbon to other species in oligotrophic desert ecosystems (Liu L. et al., 2022; Liu S. et al., 2022). This feature in particular illustrates their importance in nutrient cycling, which contributes to their dominance in the communities. These extremophiles as well as their metabolites have high thermal stability and bioavailability, which offers obvious application advantages and lays a foundation for their utilization as bioaugmentation agents in arid areas.

## Conclusion

In conclusion, we demonstrated that MGI-Tech DNBSEQ-G400 and Illumina NovaSeq 6000 platforms both reflected the composition of prokaryotic communities in the hyper-arid soil of the Turpan Basin. However, Spearman correlation analysis showed that more prokaryotic phyla identified by DNBSEQ-G400 were negatively correlated with soil physicochemical parameters, such as pH, available nitrogen, soluble salt, and soil moisture. In addition, Sloan neutral model revealed that the prokaryotic community assemblages revealed by the DNBSEQ-G400 platform were well described by neutral-based models, with relatively high coefficient fit. These results showed that the DNBSEQ-G400 could even better illustrate the diversity of prokaryotic communities in hyper-arid environments because of the good repeatability and reasonable features of the resulting assembly. Therefore, the DNBSEQ-G400 sequencing platform was further used to analyze the prokaryotic diversity at three typical hyper-arid sites, Flaming Mountain, Toksun, and Kumtag. A total of 36 prokaryotic phyla were identified across all samples, among which the eight dominant phyla were *Pseudomonadota*, *Bacteroidota*, *Bacillota*, *Actinomycetota*, *Methanobacteriota*, *Acidobacteriota*, *Nitrososphaerota*, and *Planctomycetota*. Prokaryotic community composition showed no significant differences between the three sites, which was strongly correlated with environmental factors. In addition, Sloan neutral model and β-nearest taxon index (βNTI) indicated that prokaryotic community assembly in hyper-arid areas was driven by stochastic process. Functional annotation of the Prokaryotic community showed that carbohydrate metabolism, membrane transport, and amino acid metabolism were the main metabolic pathways at three regions. Finally, phylogenetic analysis revealed that the phylum *Actinomycetota* represented a considerable level of taxonomic diversity. Overall, this work will lay a solid foundation for understanding the prokaryotic diversity and exploiting prokaryotic resources in hyper-arid areas.

## Data availability statement

The data that support the findings of this study have been deposited in China National GeneBank Sequence Archive (CNSA) of China National GeneBank DataBase (CNGBdb) with accession number CNP0004923.

## Author contributions

JC and LJ: conceptualization. ZZ and JC: methodology. ZZ and JZ: validation. JZ and YW: formal analysis and investigation. ZZ and YW: writing—original draft preparation. YW and OG: writing—review and editing. ZZ and LJ: funding acquisition. All authors contributed to the article and approved the submitted version.
