# Supplementary material for: Prokaryotic taxonomy and functional diversity assessment of different sequencing platform in a hyper-arid Gobi soil in Xinjiang Turpan Basin, China
Source: Front Microbiol. 2023 Nov 14;14:1211915. doi: 10.3389/fmicb.2023.1211915 (PMC10682777; doi:10.3389/fmicb.2023.1211915)
Supplement: Supplementary file 2 [file Data_Sheet_1.docx]

**Prokaryotic taxonomy and functional diversity assessment of different sequencing platform in a hyper-arid Gobi soil in Xinjiang Turpan Basin, China**

Zhidong Zhang^1^, Jing Zhu^1^, Osman Ghenijan^1^, Jianwei Chen^2*^, Yuxian Wang^3*^, Ling Jiang^3,4*^

^1^Institute of Applied Microbiology, Xinjiang Academy of Agricultural Sciences/ Xinjiang Key Laboratory of Special Environmental Microbiology, Urumqi, China

^2^BGI Research, Qingdao, China

^3^College of Food Science and Light Industry, Nanjing Tech University, Nanjing, China

^4^State Key Laboratory of Materials-Oriented Chemical Engineering, Nanjing Tech University, Nanjing, China

*Correspondence:

Jianwei Chen, Email: chenjianwei@genomics.cn

Yuxian Wang, Email: yxwang@njtech.edu.cn

Ling Jiang, Email: jiangling@njtech.edu.cn

**1. Figures**


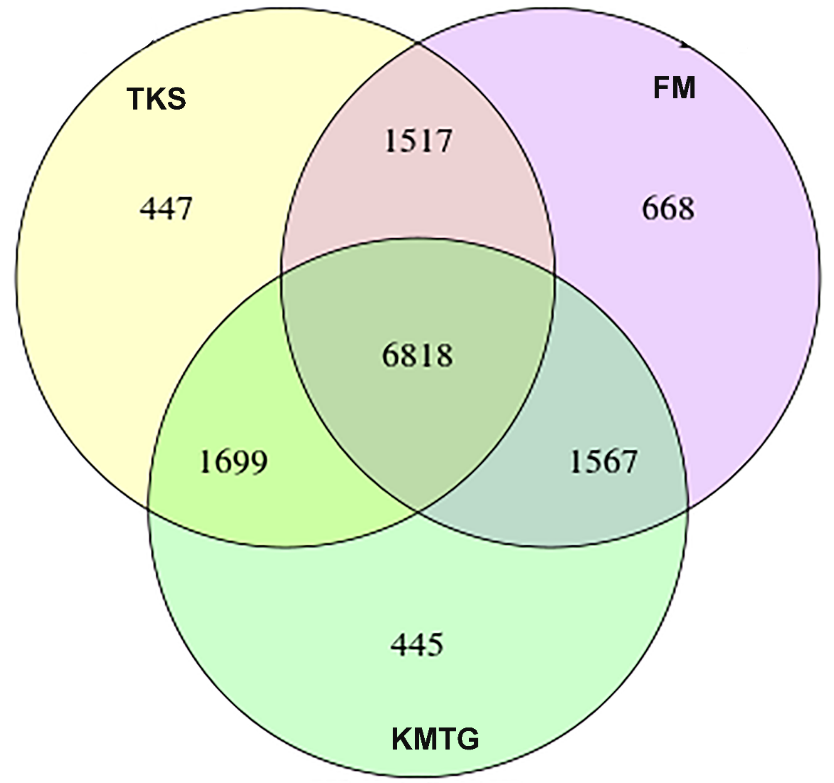


Figure S1 Shared ZOTUs across different groups.


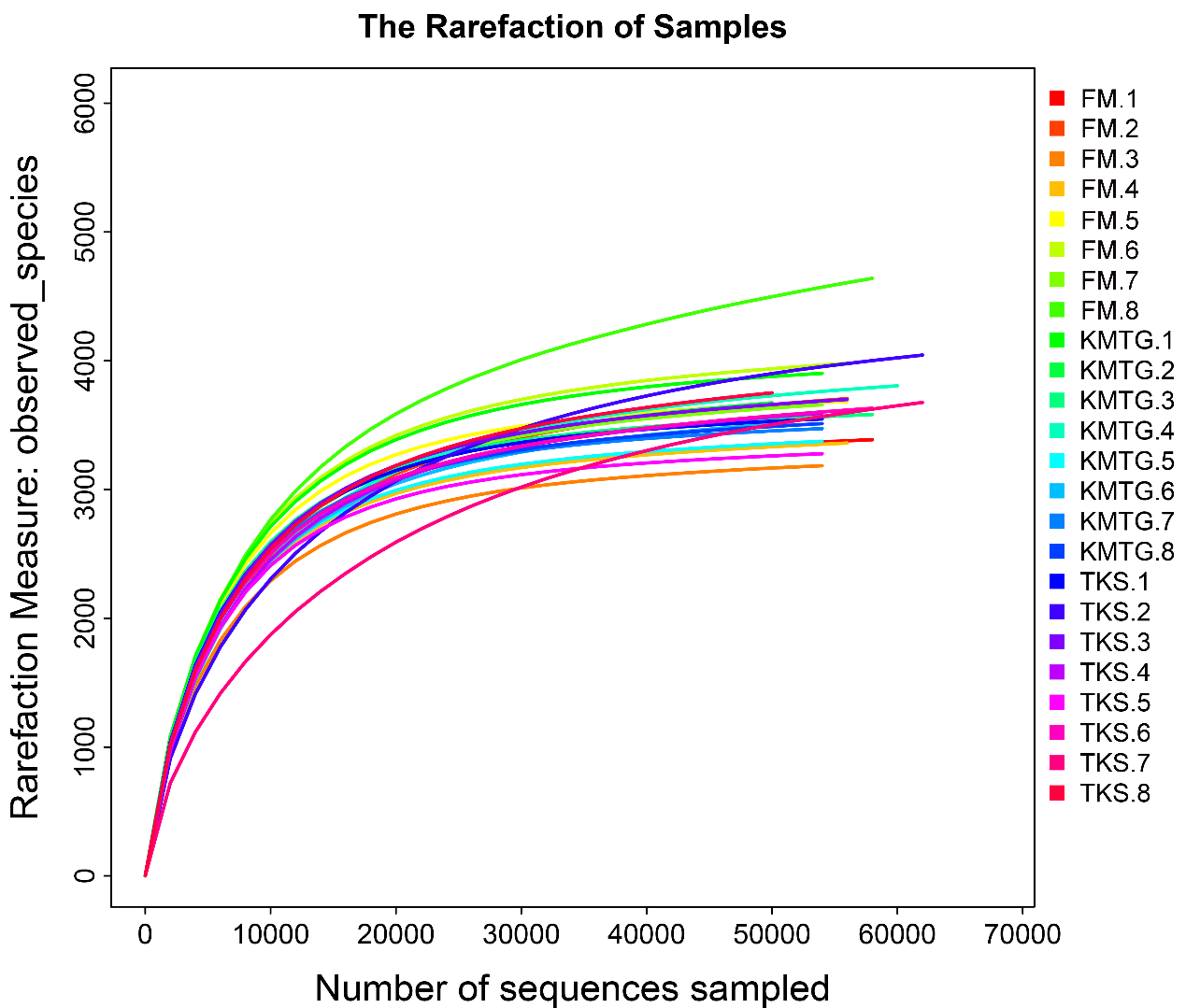


Figure S2 The rarefaction curves of α-diversity.


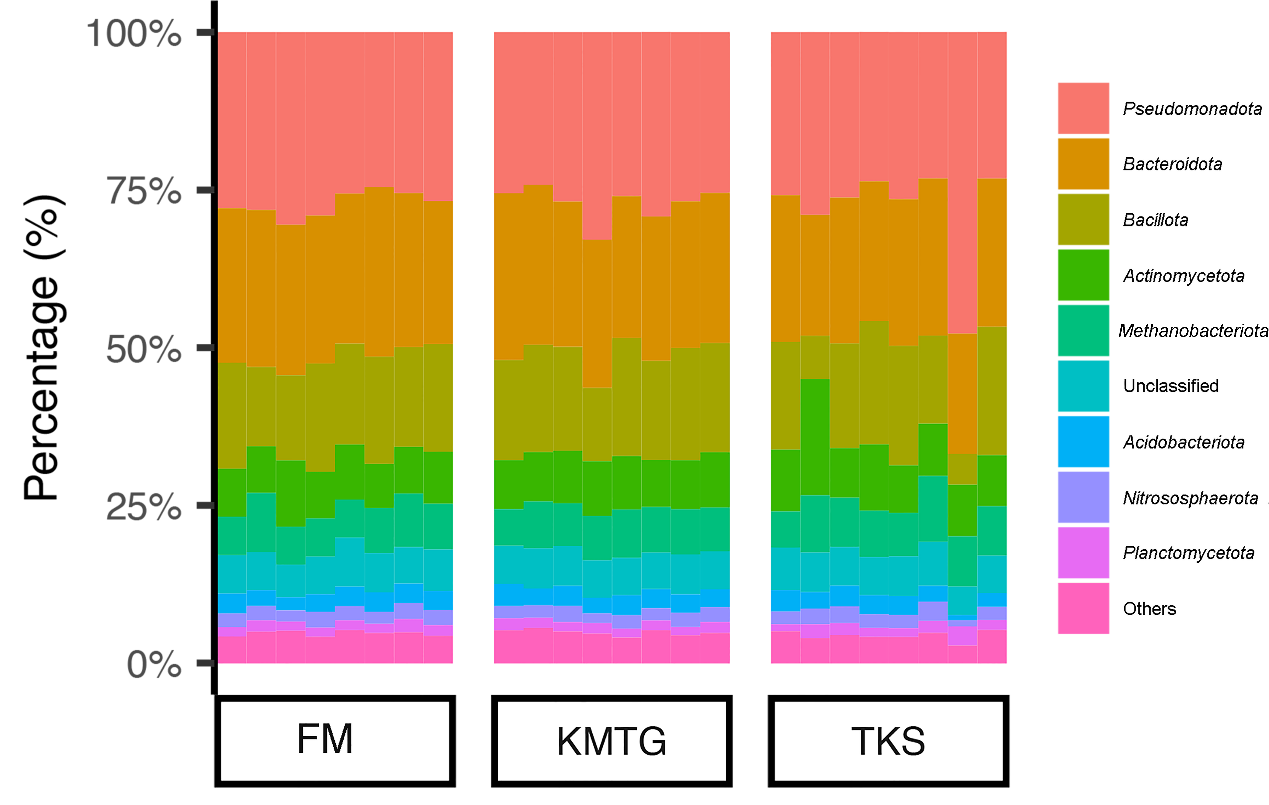


Figure S3 Abundance of the top eight taxa at phylum level.


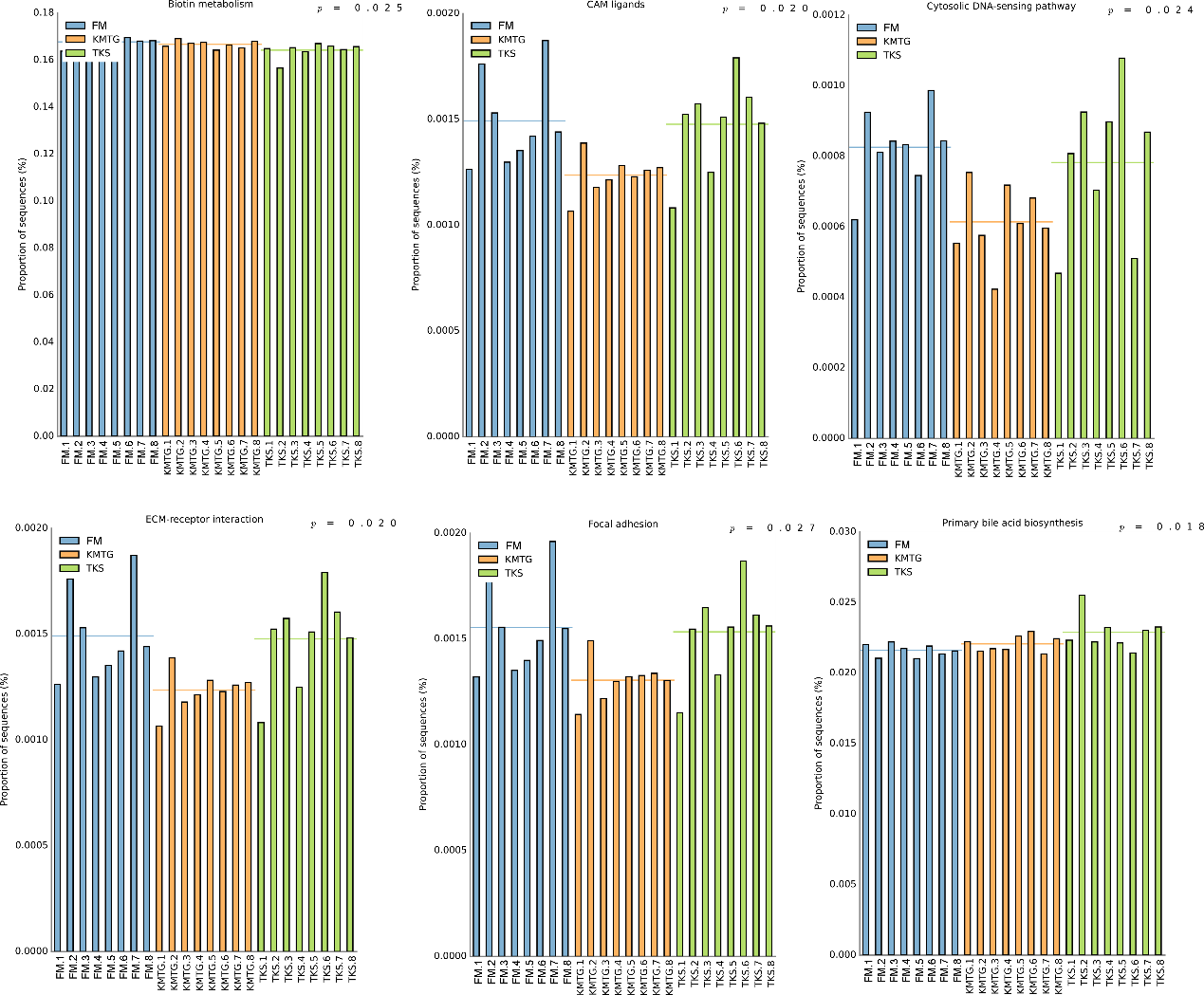


Figure S4 Pathways with significant differences between FM, KMTG, and TKS.


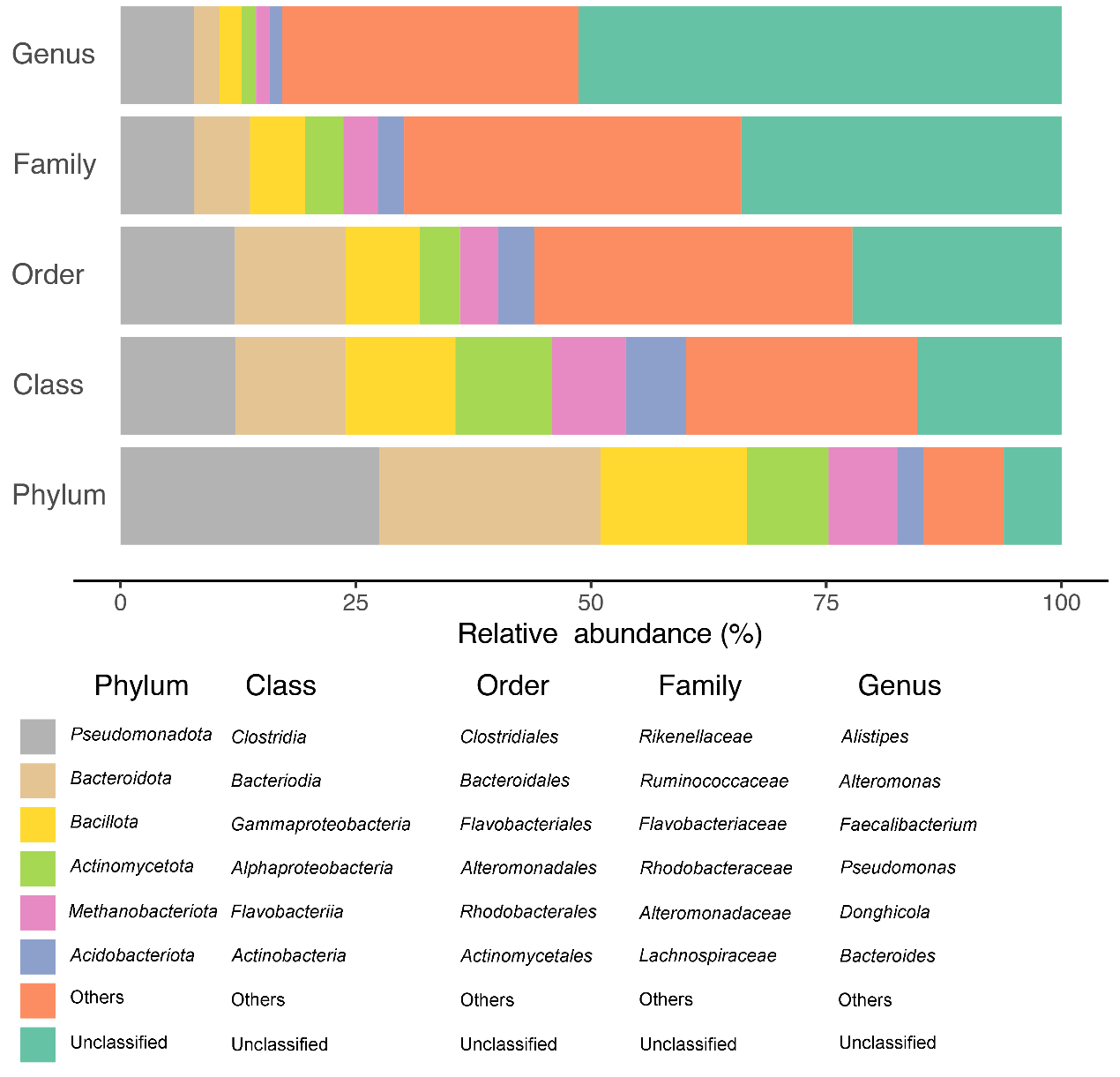


Figure S5 Relative abundance of the top six taxa from phylum to genus level.
